# Association Between Multi-Dimensional Sleep Health and Breakfast Skipping in Japanese High School Students

**DOI:** 10.3390/nu17183005

**Published:** 2025-09-19

**Authors:** Suzune Nagao, Yuh Sasawaki, Hitoshi Inokawa, Nobuko Kitagawa, Naoyuki Takashima, Kazuhiro Yagita

**Affiliations:** 1 Department of Physiology and Systems Bioscience, Graduate School of Medical Sciences, Kyoto Prefectural University of Medicine, 465 Kajii-cho, Kawaramachi-Hirokoji, Kamigyo-ku, Kyoto 602-8566, Japansasawaki@koto.kpu-m.ac.jp (Y.S.); inokawa@g.cjc.ac.jp (H.I.); nobuko-s@koto.kpu-m.ac.jp (N.K.); 2 Department of Epidemiology and Community Health and Medicine, Graduate School of Medical Sciences, Kyoto Prefectural University of Medicine, 465 Kajii-cho, Kawaramachi-Hirokoji, Kamigyo-ku, Kyoto 602-8566, Japan; n-taka@koto.kpu-m.ac.jp

**Keywords:** adolescents, daytime sleepiness, breakfast skipping, sleep health, sleep quality

## Abstract

**Background/Objectives**: Breakfast skipping has been associated with a wide range of adverse health outcomes, including metabolic disorders, disrupted circadian rhythm, and impairments of memory and attention in adolescents and adults. Although partial associations between sleep and breakfast behaviors have been reported, few studies have examined multi-dimensional sleep health simultaneously in relation to breakfast skipping, especially comprehensive studies systematically examining this relationship, particularly under controlled social conditions, remain insufficient. **Methods**: We here demonstrate the association between sleep health and breakfast skipping among 2969 Japanese high school students. Participants provided between one and eight days of sleep diary data, including meal timing records; most (78.1%) completed all eight days, while the remainder contributed fewer days. Additionally, the Pittsburgh Sleep Quality Index (PSQI) was used to assess sleep quality, and the Pediatric Daytime Sleepiness Scale (PDSS) was used to evaluate daytime sleepiness. **Results**: Later wake-up times, lower sleep quality, and stronger daytime sleepiness were each associated with a higher likelihood of breakfast skipping. In additional analyses, no significant pairwise interactions were detected among wake-up time, PSQI, and PDSS, suggesting that these factors may be separately associated with breakfast skipping. **Conclusions**: These findings suggest that multi-dimensional sleep health, including wake-up time, sleep quality, and daytime sleepiness, is relevant to breakfast skipping. This study offers a novel contribution by linking multiple downstream indicators influenced by sleep health to breakfast behavior.

## 1. Introduction

Breakfast consumption has long been recognized as an important determinant of health and learning in youth. Adolescence is a developmental period during which breakfast habits may be particularly consequential. Compared with adults, adolescents experience rapid biological and psychosocial changes while facing constraints such as early school start times. Large-scale studies have suggested that adolescents who skip breakfast may be more likely to experience poorer academic achievement [1], metabolic alterations such as prediabetes [2], and overweight or obesity, particularly when combined with insufficient sleep [3]. In addition, lower breakfast frequency has been associated with higher odds of depressive and anxiety symptoms in a large sample of Chinese adolescents [4]. Collectively, these findings indicate that in adolescence, breakfast omission may be associated with a range of cognitive, metabolic, and psychological outcomes.

At the same time, some evidence cautions against interpreting breakfast omission solely as a harmful behavior. In Norwegian high school students, common reasons for skipping breakfast included lack of time, appetite, or health-related issues, suggesting that omission may also reflect contextual or personal factors [5]. Similarly, US data indicated that students with persistent feelings of sadness or hopelessness were more likely to skip breakfast, highlighting the role of underlying psychological distress [6]. Therefore, breakfast behaviors in adolescence should be interpreted with caution, considering both potential risks and contextual explanations.

Sleep health represents another important lifestyle factor related to breakfast habits. Adolescents are recommended to obtain 8–10 h of sleep per night [7], yet many fail to do so due to delayed circadian timing and early school schedules. Studies have suggested that shorter weekday sleep and greater morning tiredness are associated with a lower likelihood of breakfast consumption [8], while in Japan, social jetlag and irregular eating patterns have been linked to poorer sleep quality and greater daytime sleepiness [9]. Circadian research has also suggested that irregular meal timing, including breakfast omission, could influence peripheral clocks and sleep regulation, although direct evidence in adolescents is limited [10].

Taken together, breakfast behavior and sleep health appear to be interconnected and may be associated with outcomes relevant to adolescent well-being. However, most prior studies have examined only single sleep dimensions or relied on adult populations. Comprehensive, multi-dimensional assessments of sleep—including timing, quality, and daytime sleepiness—in relation to breakfast behaviors remain scarce in adolescents. To address this gap, we analyzed data from the Kyoto Prefectural Lifelong Health and Medical Lifestyle Survey, one of the largest datasets on adolescent sleep health in Japan. By investigating multiple sleep indicators in relation to breakfast skipping, we aimed to describe associations that could guide future research, including the possible role of circadian processes.

## 2. Materials and Methods

### 2.1. Participants

**Figure 1:**
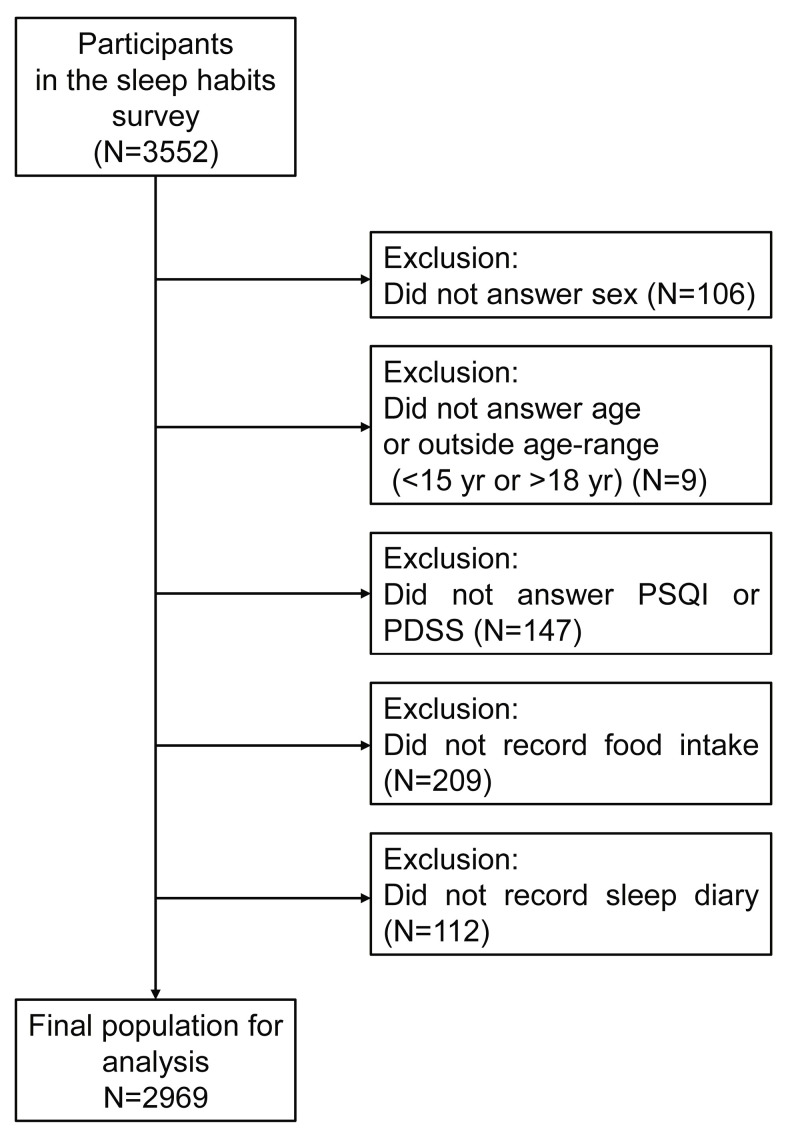
Flow diagram for inclusion and exclusion of study participants.

The data used were from a paper-based survey on sleep among Japanese high school students in Kyoto Prefecture in 2018–2019. The questionnaire consisted of age, sex, Pittsburgh Sleep Quality Index (hereinafter referred to as PSQI), Pediatric Daytime Sleepiness Scale (hereinafter referred to as PDSS), 8 days of sleep diary, and time of eating. Participants were volunteers, and responses and consent were obtained from 3552 individuals. As an exclusion condition, 106 (3.0%) participants who did not respond to sex were excluded. We then excluded 9 participants (0.3%) who reported their age to be less than 15 years old or older than 18 years old. Then, 147 (4.1%) participants with incomplete answers to the PSQI and PDSS questionnaires, 209 (5.9%) participants with no dietary records, and 112 (3.2%) participants with no sleep records were excluded in this order (Figure 1). The 2969 participants who did not meet the exclusion criteria were included in the analysis. This study was conducted in accordance with the Declaration of Helsinki and was approved by the Ethical Committee of Kyoto Prefectural University of Medicine (No. ERB-C-1340). The data that support the findings of this study are available on reasonable request from the corresponding authors. The data are not publicly available due to privacy or ethical restrictions.

### 2.2. Questionnaire

Age and sex were collected as demographic data. Sleep quality was assessed using the Japanese version of PSQI [11,12], and daytime sleepiness was evaluated using the Japanese version of PDSS [13,14]. The PSQI consists of 19 self-reported questions assessing sleep quality and disturbances over the past month. The total PSQI score, ranging from 0 to 21, is calculated based on seven components: subjective sleep quality, sleep latency, sleep duration, habitual sleep efficiency, sleep disturbances, use of sleep medication, and daytime dysfunction. A PSQI score ≥ 6 has been reported to distinguish poor sleepers from good sleepers with 89.6% sensitivity and 86.5% specificity [11]. The PDSS comprises eight items related to daytime sleepiness, each scored on a 5-point scale (0–4), with a total score ranging from 0 to 32 [13,14]. Details of all sleep-related parameters, including sleep quality, daytime sleepiness, bedtime, wake-up time, sleep duration, and breakfast consumption, are summarized in Supplementary Table S1.

### 2.3. Sleep Diary

Participants recorded sleep diaries for up to eight days, noting their bedtime, wake-up time, and meal timing (breakfast, lunch, dinner, and snacks).

### 2.4. Statistical Analysis

Breakfast skipping, the primary outcome variable in this study, was defined a priori as missing breakfast at least once during the recording period (first meal consumed before 12:00 classified as breakfast). In this study, 2775 participants (78.1%) provided complete food and sleep records for eight consecutive days. Participants were included regardless of the number of recording days to maintain statistical power. Accordingly, those who missed breakfast at least once during the recording period were categorized as the “breakfast skipping” group, while those who consumed breakfast every recorded day were categorized as the “breakfast consumption” group. For wake-up time and bedtime, the mean values of the recorded individual data were used. Sleep duration was defined as the difference between bedtime and wake-up time.

In between-group comparisons of basic characteristics (age, sex, PSQI score, PDSS score, wake-up time, bedtime, and sleep duration), categorical variables were analyzed using the chi-square test, while continuous variables were analyzed using the Wilcoxon rank-sum test. A general significance level of *p* < 0.05 was applied throughout the study unless otherwise specified.

To examine the association between breakfast skipping and sleep health components, wake-up time, bedtime, and sleep duration were each categorized into six groups. The number of participants in the breakfast skipping and breakfast consumption within each category was compared using the chi-square test. Categorization was performed by clustering wake-up time, bedtime, and sleep duration into six groups based on the median and quartiles of the data, with a granularity of 30 min. This classification aimed to clearly represent the center of the distribution and ensure data balance. Furthermore, by setting the interval width at 30 min, we aimed to facilitate intuitive and comparable interpretation of the analysis results. Specifically, wake-up time was categorized into six groups: before 6:00, 6:00–6:30, 6:30–7:00, 7:00–7:30, 7:30–8:00, and after 8:00. Bedtime was categorized into six groups: before 23:00, 23:00–23:30, 23:30–0:00, 0:00–0:30, 0:30–1:00, and after 1:00. Sleep duration was categorized into six groups: less than 6.0 h, 6.0–6.5 h, 6.5–7.0 h, 7.0–7.5 h, 7.5–8.0 h, and more than 8.0 h. As multiple comparisons were conducted in the chi-square tests involving wake-up time, bedtime, and sleep duration, Bonferroni correction was applied to control for type I error inflation, and the significance level was set at *p* < 0.0167.

Additionally, to visualize the interrelationships among sleep health components and understand the overall trend of the data, scatter plots were created for wake-up time and sleep duration, bedtime and wake-up time, and sleep duration and bedtime. Spearman’s rank correlation coefficients were calculated for these variables.

Multivariable logistic regression analysis was conducted to evaluate the relationship between breakfast consumption and age, sex, wake-up time, sleep duration, PSQI, and PDSS. Age was categorized into three groups: 15 years (reference), 16 years, and 17–18 years. Sex was categorized into two groups: male (reference) and female. Wake-up time was categorized into six groups: before 6:00 (reference), 6:00–6:30, 6:30–7:00, 7:00–7:30, 7:30–8:00, and after 8:00. Sleep duration was categorized into six groups: less than 6.0 h (reference), 6.0–6.5 h, 6.5–7.0 h, 7.0–7.5 h, 7.5–8.0 h, and more than 8.0 h. PSQI was categorized into two groups: <6 (reference) and ≥6. PDSS was categorized into two groups: <21 (reference) and ≥21.

The adjusted odds ratio (aOR) and 95% confidence interval (95% CI) were calculated for each variable (hereinafter referred to as aOR and 95% CI, respectively). Statistical significance was determined at the standard threshold unless otherwise specified. Model goodness-of-fit was evaluated using the Akaike Information Criterion (AIC) and Bayesian Information Criterion (BIC) (hereinafter referred to as AIC and BIC, respectively), while variance inflation factors (VIF) were used to assess multicollinearity (hereinafter referred to as VIF). In this study, all explanatory variables selected based on the study objectives were included in the model, allowing us to statistically evaluate the overall influence of these variables and test the research hypotheses.

To assess the association between breakfast consumption and sleep quality or daytime sleepiness, the cutoff values for PSQI and PDSS were set at 6 and 21 points, respectively, based on previous studies [11,14]. The ratio of participants in the breakfast consumption to those in the breakfast skipping was calculated, and a chi-square test was performed.

Stacked bar charts were generated using Jupyter (version 6.0.3), multivariable logistic regression analysis was conducted using JMP Pro (version 18.1.0), and scatter plots and pie charts were generated using Origin Pro 2024 (version 10.1.0.178).

In addition to the primary definition (Breakfast consumption vs. Breakfast skipping), we performed sensitivity analyses using an alternative definition (Any breakfast consumption vs. complete skipping). These analyses are presented in Supplementary Tables S2 and S3.

## 3. Results

### 3.1. Baseline Characteristics

Table 1 shows the baseline characteristics of the 2969 subjects. There was a significant difference in age composition between breakfast consumption (N = 1850) and breakfast skipping (N = 1119) (*p* < 0.001), with a trend toward older age in breakfast skipping. There was no significant difference in sex, and the scores of PSQI and PDSS were significantly higher in the skipping meal group than in breakfast consumption group. Both wake-up time and bedtime were significantly later in breakfast skipping than in breakfast consumption. Sleep duration was significantly longer in breakfast skipping than in breakfast consumption (Table 1). Sensitivity analyses using the alternative definition (any breakfast consumed vs. complete skipping) produced broadly similar patterns of association, including no significant interactions among sleep indicators (see Supplementary Table S2).

### 3.2. Relationship Between Breakfast Skipping Rate and Dimensions of Sleep Health

**Figure 2:**
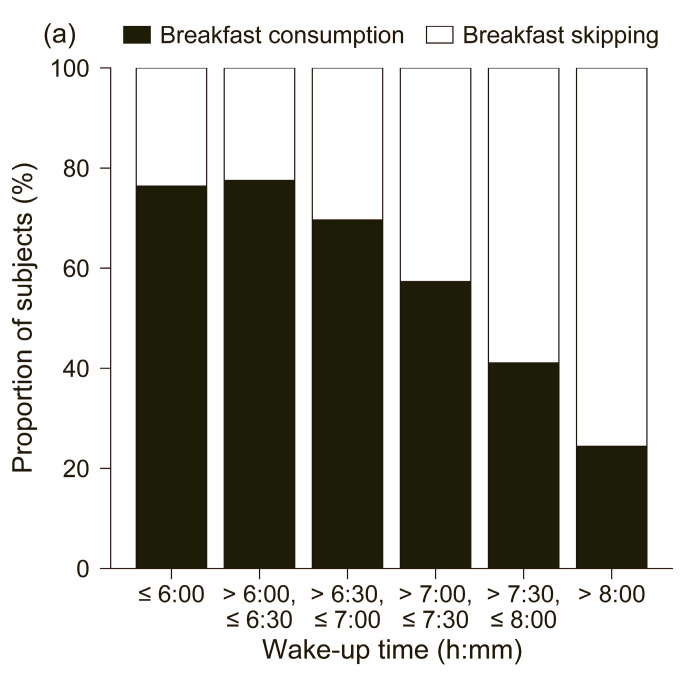

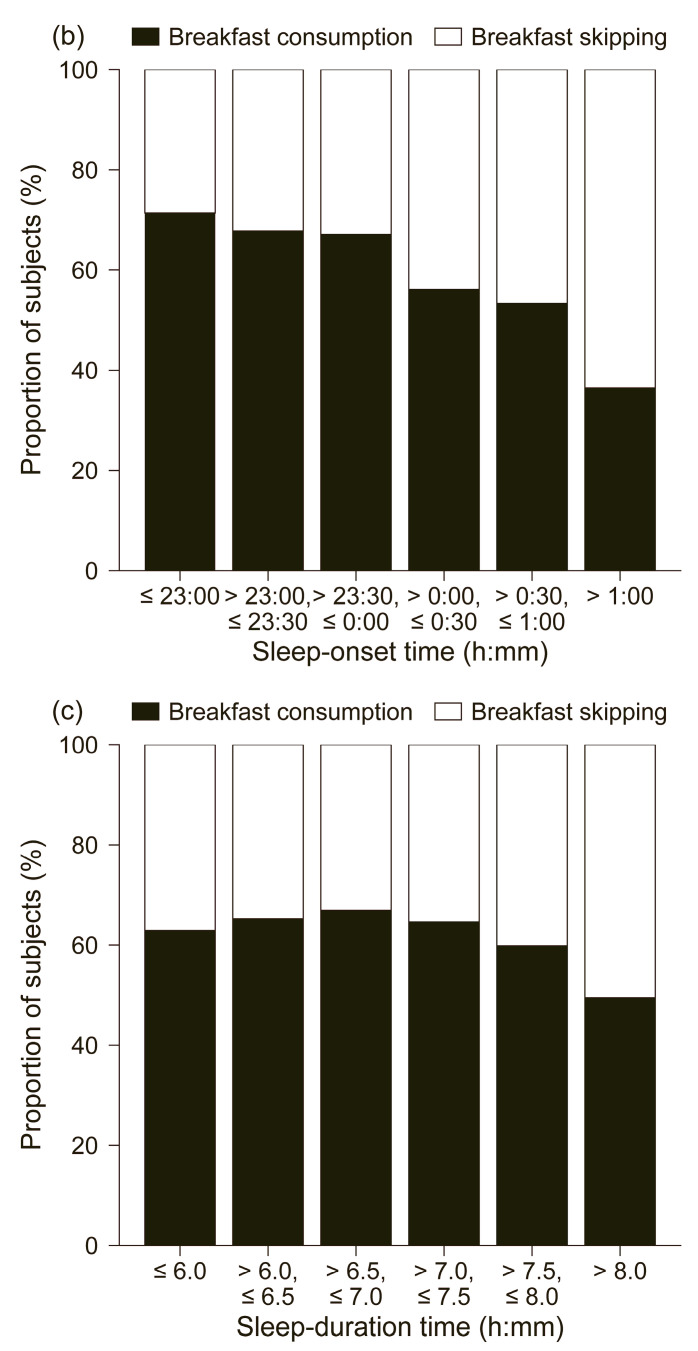
Relationship between breakfast skipping rate and (**a**) wake-up time, (**b**) bedtime, and (**c**) sleep duration.

Figure 2 shows the number ratios of breakfast consumption and breakfast skipping by wake-up time, bedtime, and sleep duration. The breakfast rate was 23.6% in the group wake-up before 6:00 (N = 550), while it was 30.3% in the group wake-up between 6:30 and 7:00 (N = 685), 42.6% in the group wake-up between 7:00 and 7:30 (N = 598), and 75.6% in the group wake-up after 8:00 (N = 279) The results of the chi-square test showed that there was a statistically significant difference between the breakfast skipping rate and the time of wake-up (χ^2^ = 355.2, *p* = 1.4 × 10^−74^), suggesting that the breakfast skipping rate increases as the time of wake-up becomes later. In terms of bedtime, the rate of breakfast skipping was 28.6% in the group who went to bed before 23:00 (N = 665), 32.9% in the group who went to bed between 23:30 and 0:00 (N = 836), 44.0% in the group who went to bed between 0:00 and 0:30 (N = 417) and the group who went to bed after 1:00 (N = 293). Using a chi-square test, we found a statistically significant difference between bedtime and breakfast skipping (χ^2^ = 138.9, *p* = 3.0 × 10^−28^), suggesting that a late bedtime is associated with breakfast skipping. In terms of sleep duration, the rate of breakfast skipping was 37.1% in the group sleeping less than 6.0 h (N = 458), 48.5% in the group sleeping 6.5–7.0 h (N = 667), 39.9% in the group sleeping 7.5–8.0 h (N = 426), and 49.5% in the group sleeping more than 8.0 h (N = 380). The results of the analysis using the chi-square test showed a statistically significant difference between the sleep duration and the breakfast skipping rate (χ^2^ = 36.6, *p* = 7.3 × 10^−7^), suggesting that the breakfast skipping rate is lowest at approximately 7 h of sleep.

### 3.3. Interrelationships Between Dimensions of Sleep Health

**Figure 3:**
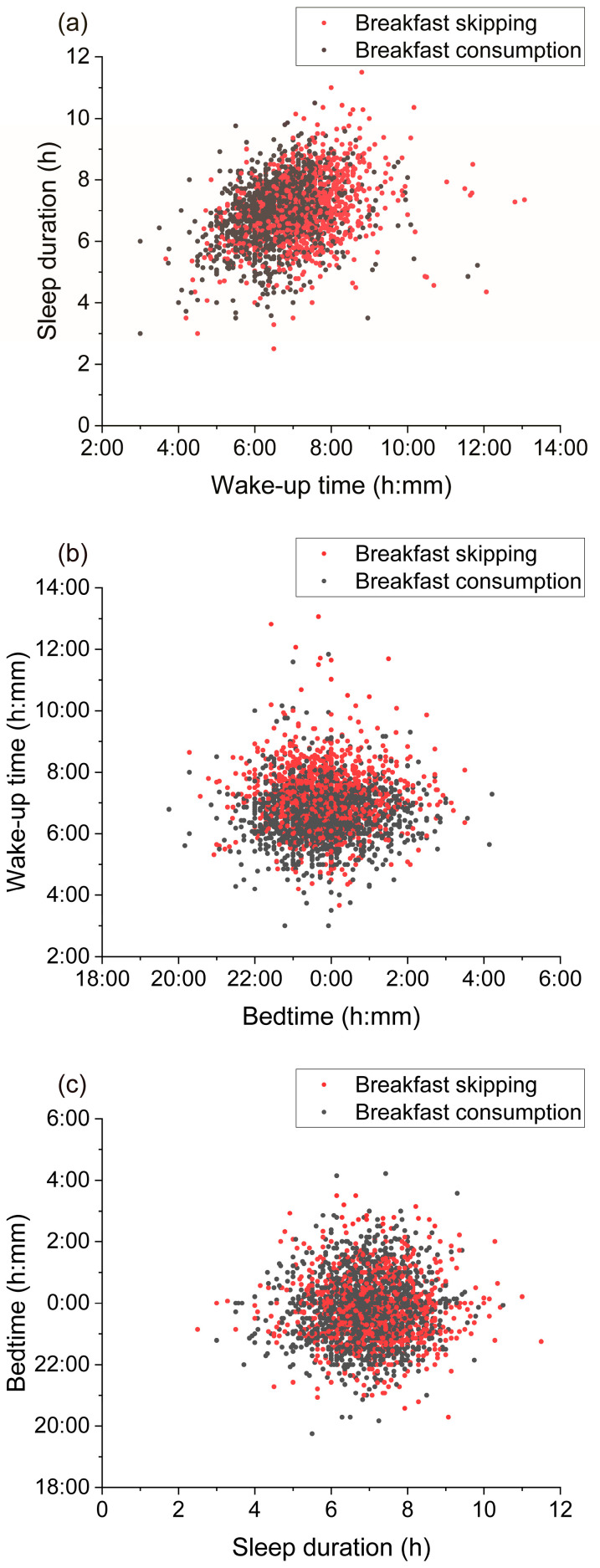
Scatter plot of (**a**) wake-up time and sleep duration, (**b**) bedtime and wake-up time, (**c**) sleep duration and bedtime.

Figure 3 shows the results of the analysis of the association between wake-up time, sleep duration and bedtime using Spearman’s rank correlation coefficient. A moderate positive correlation was found between wake-up time and sleep duration, and the association was significant (ρ = 0.3591, *p* < 0.0001, Figure 3a). No significant correlation was found between bedtime and wake-up time (ρ = −0.0011, *p* = 0.9514, Figure 3b) or between sleep duration and bedtime (ρ = 0.0012, *p* = 0.9463, Figure 3c). These results suggest that wake-up time and sleep duration are related, and that bedtime is independent of wake-up time and sleep duration.

### 3.4. Identifying Sleep Health Indicators Associated with Breakfast Skipping Using Multivariable Logistic Regression Analysis

The results of the multivariable logistic regression analysis are shown in Table 2. The goodness of fit of the model is AIC = 3537.9 and BIC = 3633.65. VIF was less than 2 for all independent variables, and no association was found between them. Age had a significant effect on the risk of breakfast skipping, with a lower likelihood of breakfast skipping in the 16-year-old and 17–18-year-old groups compared to the 15-year-old group (aOR = 1.42, 95% CI: 1.17–1.75, *p* = 0.0005, aOR = 1.39, 95% CI: 1.10–1.75, *p* = 0.0051, respectively). No significant difference was found for sex (aOR = 1.11, 95% CI: 0.94–1.30, *p* = 0.2255).

The effect of wake-up time on breakfast skipping was not significant in the group that woke up between 6:00 and 6:30 (aOR = 1.00, 95% CI: 0.75–1.35, *p* = 0.9776), was significant in the group that woke up between 7:00 and 7:30 (aOR = 1.49, 95% CI: 1.14–1.95, *p* = 0.0034), was significant in the group that woke up between 7:30 and 8:00 (aOR = 2.58, 95% CI: 1.97–3.40, *p* < 0.0001), and was significant in the group that woke up after 8:00 (aOR = 9.43, 95% CI: 6.62–13.45, *p* < 0.0001) compared to the group that woke up before 6:00. These indicate that the odds ratio increased as the wake-up time became later. In the sensitivity analysis, although PSQI was not associated with never eating breakfast, later wake-up times were associated with progressively higher odds of complete breakfast skipping, PDSS scores ≥ 21 were linked to higher odds, and sleep durations around seven hours were linked to lower odds; these patterns mirrored those observed in the primary analysis (see Supplementary Table S3). Following the sensitivity analyses, we additionally examined breakfast-pattern regularity by comparing three groups: eating breakfast every day, eating breakfast irregularly (≥1 day breakfast and ≥1 day skipping), and never eating breakfast. Wake-up time became progressively later across these groups (see Supplementary Table S4). Based on the perspective of sleep behavioral regularity, PSQI and PDSS were significantly lower in the “regular” groups (eating breakfast every day or never eating breakfast) than in the irregular group (see Supplementary Table S5), indicating better subjective sleep quality and less daytime sleepiness among students with regular breakfast patterns.

In addition, we further examined potential interaction effects among the three sleep-related variables that were independently associated with breakfast skipping—wake-up time, PSQI, and PDSS—using multivariable logistic regression models. However, none of the interaction terms were statistically significant, indicating that these variables influenced breakfast skipping independently without modifying each other’s effects. Consistent with the primary analysis, no significant interactions were detected among wake-up time, PSQI, and PDSS in the sensitivity analysis.

### 3.5. Association Between PSQI, PDSS and Breakfast Skipping

**Figure 4:**
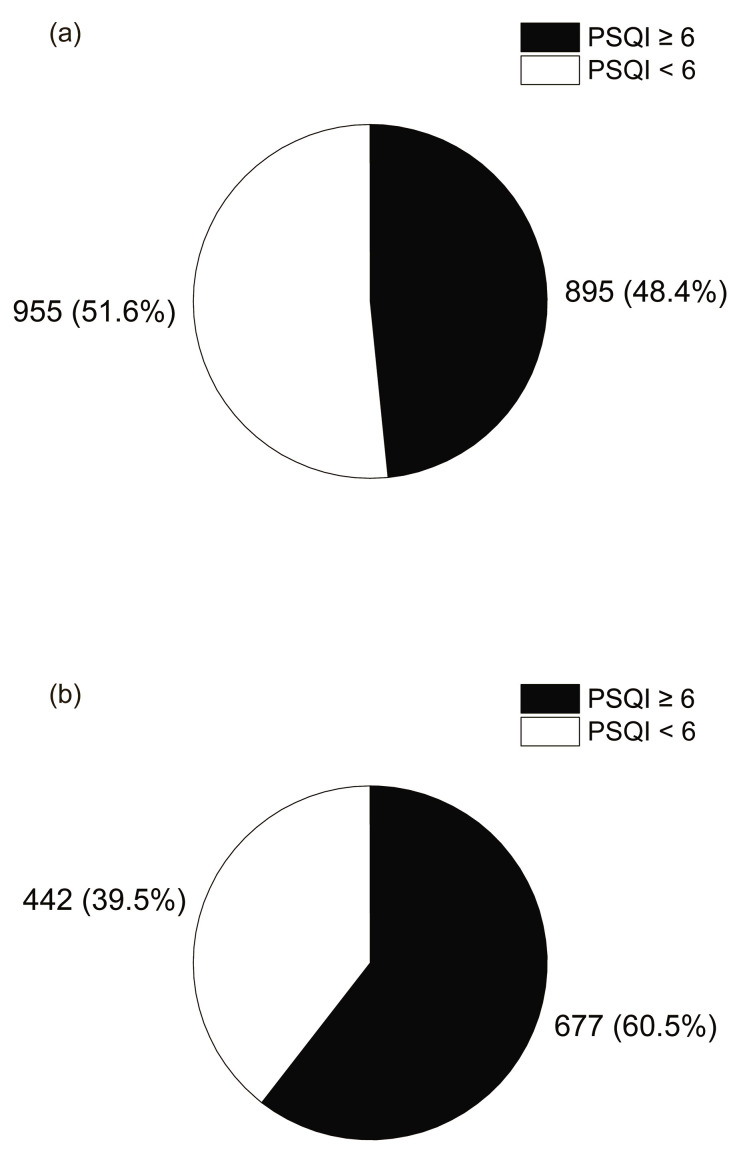
Pie chart showing the percentage of people with good or bad sleep quality according to whether they (**a**) eat breakfast every day or (**b**) skip breakfast at least 1 day.

**Figure 5:**
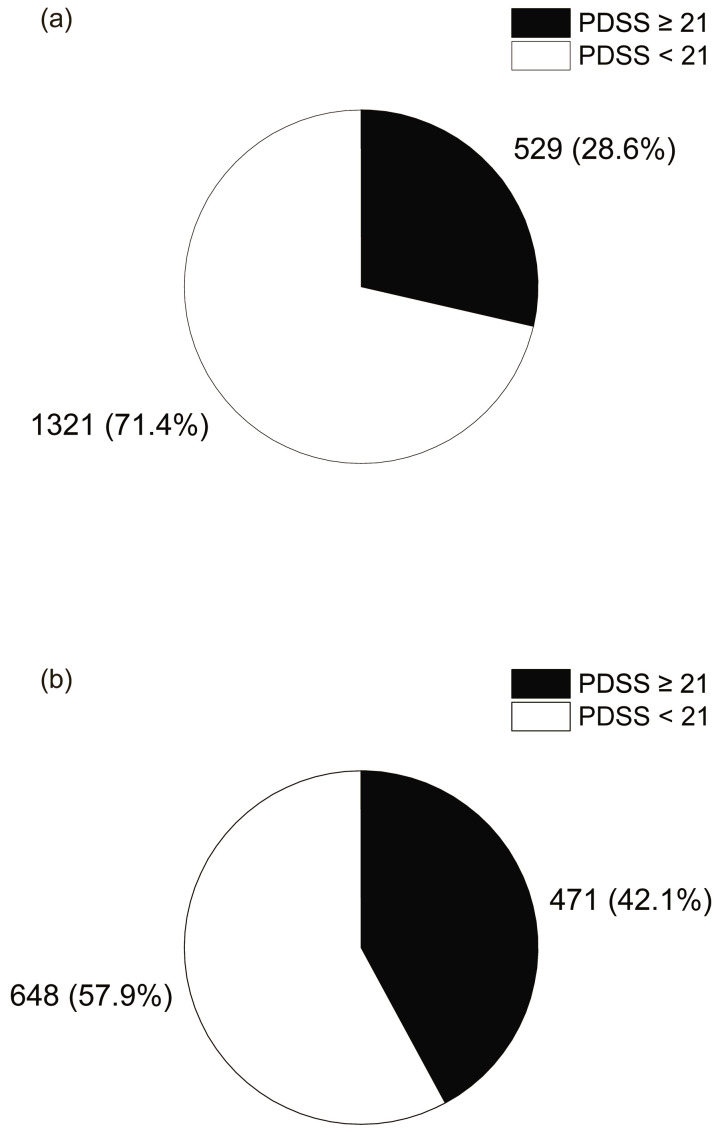
Pie chart showing the percentage of people with daytime sleepiness according to whether they (**a**) eat breakfast every day or (**b**) skip breakfast at least 1 day.

Figure 4 shows the relationship between the PSQI score, which indicates sleep quality, and the rate of breakfast skipping. The percentage of breakfast skipping was 60.5% in the group with a PSQI score above the cutoff of 6, which was significantly higher in the chi-square test compared to 48.4% in the group with a PSQI score below the cutoff of less than 6 (*p* < 0.001). This result suggests that poor sleep quality is associated with breakfast skipping. Figure 5 shows the relationship between the PDSS score, which indicates daytime sleepiness, and the rate of breakfast skipping. The breakfast skipping rate was 57.9% in the group with a PDSS score ≥ 21, which was significantly higher than that in the group with a PDSS score < 21 (28.6%) (*p* < 0.001). Breakfast skipping was significantly correlated with greater daytime sleepiness, suggesting the presence of an associated pattern.

## 4. Discussion

This study explored the associations between multi-dimensional sleep health and breakfast skipping among Japanese high school students, using a large dataset and multiple analytical approaches. In the primary analysis, later wake-up times, lower sleep quality, and higher daytime sleepiness were each independently associated with a greater likelihood of breakfast skipping. Consistent with this, sensitivity analyses using an alternative definition of breakfast skipping (any breakfast consumption vs. complete skipping) produced broadly similar patterns—including rising odds with later wake-up times, higher PDSS scores, and lower odds near seven hours of sleep duration (see Supplementary Tables S2 and S3). In both the primary and sensitivity analyses, no significant interaction effects were detected among wake-up time, PSQI, and PDSS, suggesting these sleep dimensions may independently influence breakfast behavior.

These findings align with prior evidence showing that adolescents with poorer overall sleep health are less likely to consume breakfast regularly [15]. They also resonate with research indicating that breakfast skipping is more common among adolescents with poorer emotional wellbeing [16], supporting the idea that breakfast behavior may reflect underlying contextual or physiological factors rather than simply an unhealthy habit. Taken together, these findings suggest that breakfast skipping may reflect misalignments between internal circadian timing and socially imposed schedules, rather than solely unhealthy habits. Building on our sensitivity analyses, we further examined breakfast-pattern regularity. In these supplementary analyses, wake-up times became progressively later across the three groups (daily breakfast < irregular breakfast < daily skipping), and both earlier and more stable wake-up times and significantly lower PSQI and PDSS scores were observed in the “regular” groups (daily breakfast or daily skipping) compared with the irregular group. These findings suggest that behavioral regularity—rather than breakfast consumption per se—may be an important factor associated with more favorable sleep timing and better subjective sleep health in adolescents.

Although our cross-sectional design limits causal inference, the consistency across definitions suggests a stable association of sleep dimensions with breakfast behavior. These parallel patterns across sleep timing, quality, and daytime functioning may reflect underlying circadian misalignment, which has been implicated in influencing appetite, sleep quality, and metabolic regulation—though evidence in adolescents remains limited.

This study has several limitations. First, its cross-sectional design precludes causal inference. Second, the reliance on self-reported measures may have introduced recall or reporting bias. Third, the sample was limited to Japanese high school students, which restricts generalizability to other populations.

Despite these limitations, the consistent findings across multiple operational definitions—including various sleep indicators and sensitivity analyses—enhance confidence in the robustness of the key message. By linking multiple dimensions of sleep health to breakfast behavior, this study provides a foundation for future longitudinal and experimental research aimed at clarifying underlying mechanisms and health trajectories.

In future research, randomized school-based programs that promote regular breakfast or target sleep hygiene may elucidate whether improvements in breakfast habits lead to better metabolic markers or wellbeing outcomes, supporting causal inference.

## 5. Conclusions

The present study suggests that breakfast skipping in high school students is associated with multi-dimensional sleep health, particularly with later wake-up times, lower sleep quality, and stronger daytime sleepiness. These associations remained consistent in sensitivity analyses using an alternative definition of breakfast skipping, and no significant interactions among the main sleep indicators were detected, suggesting that each factor may independently influence breakfast behavior. This pattern may reflect not only behavioral habits but also potential circadian misalignment between internal biological timing and social schedules. Our findings highlight the need to consider multiple aspects of sleep when addressing breakfast skipping in adolescents. Future studies should adopt longitudinal and interventional designs, include objective biological markers such as glucose and other metabolic or hormonal parameters, and examine broader age groups—including individuals with obesity or type 2 diabetes—to clarify causal pathways and inform targeted prevention strategies.

## Figures and Tables

**Table 1:** Comparison of baseline characteristics and dimensions of sleep health classified by breakfast skipping status.

Characteristic		Subjects (N = 2969)	Breakfast Consumption (N = 1850)	Breakfast Skipping (N = 1119)	*p*
Age, N.	15	754	512	242	<0.001
	16	1441	864	577	
	17	767	468	299	
	18	7	6	1	
Sex, N.	Male	1368	862	506	0.466
	Female	1601	988	613	
PSQI, means ± SD		5.8 ± 2.4	5.5 ± 2.2	6.3 ± 2.5	<0.001
PDSS, means ± SD		18.2 ± 5.1	17.6 ± 5.0	19.3 ± 5.1	<0.001
Wake-up time, means ± SD (h:min)		6:52 ± 0:58	6:38 ± 0:50	7:14 ± 1:02	<0.001
Bedtime, means ± SD (h:min)		23:47 ± 0:59	23:39 ± 0:54	00:02 ± 1:05	<0.001
Sleep duration, means ± SD (h)		7.0 ± 1.0	6.9 ± 0.9	7.1 ± 0.9	<0.001

Notes: SD, standard deviation; PSQI, Pittsburgh Sleep Quality Index; PDSS, Pediatric Daytime Sleepiness Scale.

**Table 2:** Multivariable logistic regression analysis of breakfast skipping with breakfast consumption as the reference category.

		aOR	95%CI	*p*
Age	(a) 15 years old	ref		
	(b) 16 years old	1.42	1.17–1.75	0.0005
	(c) 17 and 18 years old	1.39	1.10–1.75	0.0051
Sex	(a) Male	ref		
	(b) Female	1.11	0.94–1.30	0.2255
Wake-up time	(a) ≤6:00	ref		
	(b) >6:00 and ≤6:30	1.00	0.75–1.35	0.9776
	(c) >6:30 and ≤7:00	1.49	1.14–1.95	0.0034
	(d) >7:00 and ≤7:30	2.58	1.97–3.40	<0.0001
	(e) >7:30 and ≤8:00	4.83	3.54–6.59	<0.0001
	(f) >8:00	9.43	6.62–13.45	<0.0001
Sleep duration	(a) ≤6.0 h	ref		
	(b) >6.0 h and ≤6.5 h	0.87	0.65–1.18	0.3684
	(c) >6.5 h and ≤7.0 h	0.83	0.63–1.09	0.1782
	(d) >7.0 h and ≤7.5 h	0.80	0.60–1.06	0.1199
	(e) >7.5 h and ≤8.0 h	0.86	0.63–1.17	0.3283
	(f) >8.0 h	1.07	0.78–1.48	0.6728
PSQI	(a) <6	ref		
	(b) ≥ 6	1.39	1.17–1.65	0.0001
PDSS	(a) <21	ref		
	(b) ≥ 21	1.46	1.23–1.74	0.0001

Notes: aOR, adjusted odds ratio; 95% CI, 95% confidence interval; PSQI, Pittsburgh Sleep Quality Index; PDSS, Pediatric Daytime Sleepiness Scale; ref, reference category.

## Data Availability

The data that support the findings of this study are available upon reasonable request from the corresponding authors. The data are not publicly available due to privacy and ethical restrictions.

## Supplementary Materials

The following supporting information can be downloaded at: https://www.mdpi.com/article/10.3390/nu17183005/s1, Table S1: Summary of all sleep-related parameters (quality, daytime sleepiness, bedtime, wake-up time, sleep duration) and breakfast consumption measures; Table S2: Comparison of baseline characteristics and dimensions of sleep health classified by breakfast skipping status (any breakfast consumed vs. complete skipping); Table S3: Multivariable logistic regression analysis of breakfast skipping with any breakfast consumed as the reference category; Table S4: Comparison of wake-up times by breakfast frequency; Table S5: Comparison of average sleep indices by frequency of breakfast frequency.
