# Supplementary material for: Association Between Multi-Dimensional Sleep Health and Breakfast Skipping in Japanese High School Students
Source: Nutrients. 2025 Sep 19;17(18):3005. doi: 10.3390/nu17183005 (PMC12472367; doi:10.3390/nu17183005)
Supplement: Supplementary file 1 [file nutrients-17-03005-s001.zip › nutrients-3868841-supplementary.pdf]

Supplementary Table S1. Summary of all sleep-related parameters (quality, daytime sleepiness, bedtime, wake-up time, sleep duration) and breakfast consumption measures.

**Measure / Parameter**	**Instrument / Source**	**Score Range / Unit**	**Cut-off (if used)**
Sleep quality	PSQI, Japanese version	Total score 0–21	≥6 = poor sleep quality
Daytime sleepiness	PDSS, Japanese version	Total score 0–32	≥21 = high daytime sleepiness
Bedtime	Self-reported sleep diary (1–8 days)	Mean of recorded days	Categorized into 6 groups: <23:00, 23:00–23:30, 23:30–0:00, 0:00–0:30, 0:30–1:00, >1:00
Wake-up time	Self-reported sleep diary (1–8 days)	Mean of recorded days	Categorized into 6 groups: <6:00, 6:00–6:30, 6:30–7:00, 7:00–7:30, 7:30–8:00, >8:00
Sleep duration	Derived from bedtime and wake-up time (1–8 days)	Mean of recorded days	Categorized into 6 groups: <6.0 h, 6.0–6.5 h, 6.5–7.0 h, 7.0–7.5 h, 7.5–8.0 h, >8.0 h
Breakfast consumption	Meal timing from same sleep diary (1–8 days)	Binary variable: consumed vs. skipped	Skipping defined as ≥1 day without breakfast during the observation period

Notes:

1. PSQI, Pittsburgh Sleep Quality Index; PDSS, Pediatric Daytime Sleepiness Scale.
2. PSQI consists of seven components: subjective sleep quality, sleep latency, sleep duration, habitual sleep efficiency, sleep disturbances, use of sleep medication, and daytime dysfunction.
3. PDSS comprises eight items related to daytime sleepiness.
4. Most participants (78%) completed all eight days of diary entries; others contributed fewer days.
5. Categorization for bedtime, wake-up time, and sleep duration was performed as described in the Methods.

Supplementary Table S2. Comparison of baseline characteristics and dimensions of sleep health classified by breakfast skipping status (any breakfast consumed vs. complete skipping)

Characteristic		Subjects (N=2969)	Any breakfast consumed (N=2884)	Complete skipping (N=85)	p
Age, n.	15	754	734	20	0.597
	16	1441	1394	47	
	17	767	749	18	
	18	7	7	0	
Sex, n.	Male	1368	1319	49	0.030
	Female	1601	1565	36	
PSQI, means ±SD		5.8 ±2.4	5.8±2.3	6.5±2.7	0.022
PDSS, means ±SD		18.2 ±5.1	18.2±5.1	19.5±5.4	0.027
Wake-up time, means ±SD (h:min)		6:52 ±0:58	6:50±0:57	7:29±0:56	<0.001
Bedtime, means ±SD (h:min)		23:47±0:59	23:47±0:59	23:52±0:55	0.511
Sleep duration, means ±SD (h)		7.0 ±1.0	7.0±1.0	7.1±1.3	0.481

Notes: SD, standard deviation; PSQI, Pittsburgh Sleep Quality Index; PDSS, Pediatric Daytime Sleepiness Scale. P-values were obtained using independent samples t-tests (two-tailed) for pairwise comparisons, with significance set at $p < 0.05$.

Supplementary Table S3. Multivariable logistic regression analysis of breakfast skipping with any breakfast consumed as the reference category

		aOR	95%CI	p
Age	a) 15 years old	ref		
	b) 16 years old	1.17	0.68-2.01	0.5745
	c) 17 and 18 years old	0.82	0.42-1.59	0.5541
Sex	a) Male	ref		
	b) Female	0.61	0.40-0.95	0.0299
Wake-up time	a) ≤6:00	ref		
	b) >6:00 and ≤6:30	2.18	0.72-6.64	0.1687
	c) >6:30 and ≤7:00	3.32	1.19-9.22	0.0216
	d) >7:00 and ≤7:30	3.81	1.36-10.63	0.0107
	e) >7:30 and ≤8:00	4.71	1.61-13.76	0.0046
	f) >8:00	9.82	3.54-27.25	<.0001
Sleep duration	a) ≤6.0 h	ref		
	b) >6.0 h and ≤6.5 h	0.62	0.28-1.38	0.2442
	c) >6.5 h and ≤7.0 h	0.46	0.22-0.99	0.0480
	d) >7.0 h and ≤7.5 h	0.53	0.25-1.12	0.0952
	e) >7.5 h and ≤8.0 h	0.52	0.23-1.16	0.1100

	f) >8.0 h	0.72	0.34-1.54	0.3998
PSQI	a) <6	ref		
	b) ≥6	0.98	0.61-1.58	0.9437
PDSS	a) <21	ref		
	b) ≥21	1.60	1.01-2.54	0.0468

Notes: aOR, adjusted odds ratio; 95% CI, 95% confidence interval; PSQI, Pittsburgh Sleep Quality Index; PDSS, Pediatric Daytime Sleepiness Scale; ref, reference category. "All variance inflation factors (VIFs) were below 2.5, indicating no evidence of problematic multicollinearity among independent variables.

Supplementary Table S4. Comparison of wake-up times by breakfast frequency

	**Wake-up time**	**p**
Eating breakfast every day, means ±SD (h:min)	6:38±0:50	<0.0001
Eating breakfast irregularly, means ±SD (h:min)	7:13±1:03	
Never eating breakfast means, ±SD (h:min)	7:29±0:56	

Notes: SD, standard deviation; PSQI. P-values were obtained using one-way ANOVA, conducted using JMP Student Edition 18.2.1. In all pairwise comparisons using Tukey-Kramer's HSD test, significant differences were found between all groups.

Supplementary Table S5. Comparison of average sleep indices by frequency of breakfast frequency

	**Skipping breakfast everyday or Eating breakfast everyday**	**Eating breakfast irregularly**	**p**
PSQI, means ±SD	5.5±2.2	6.3±2.4	<0.0001
PDSS, means ±SD	17.62±5.0	19.2.±5.1	<0.0001

Notes: SD, standard deviation; PSQI, Pittsburgh Sleep Quality Index; PDSS, Pediatric Daytime Sleepiness Scale. P-values were obtained using independent samples t-tests (two-tailed) for pairwise comparisons, with significance set at $p < 0.05$.
